# Lipid droplets and mitochondria are anchored during brown adipocyte differentiation

**DOI:** 10.1007/s13238-019-00661-1

**Published:** 2019-10-30

**Authors:** Liujuan Cui, Ahmed Hammad Mirza, Shuyan Zhang, Bin Liang, Pingsheng Liu

**Affiliations:** 1 School of Life Sciences, University of Science and Technology of China, Hefei, 230027 China; 2 National Laboratory of Biomacromolecules, CAS Center for Excellence in Biomacromolecules, Institute of Biophysics, Chinese Academy of Sciences, Beijing, 100101 China; 3 University of Chinese Academy of Sciences, Beijing, 100049 China; 4 State Key Laboratory of Conservation and Utilization of Bio-Resources in Yunnan, and Center for Life Sciences, School of Life Sciences, Yunnan University, Kunming, 650091 Yunnan China

**Dear Editor,**

Brown adipose tissue (BAT) acts as a site of non-shivering thermogenesis in mammals including adult humans (Nedergaard and Cannon, 2010). Prolonged cold exposure can induce the acquisition of thermogenic function in white adipose tissue (WAT) with the cells that have undergone the “browning” process being referred to as beige cells (Wu et al., 2013). Both mainly use mitochondrial β-oxidation of fatty acid (FA) from lipid droplets (LDs) in maintaining body temperature. The efficiency of hydrophobic FA transport between two organelles in aqueous cytoplasm has been challenged. Understanding the mechanism underlying the interaction between these two organelles is essential for animal physiology as well as for the development of therapies to treat metabolic diseases (Benador et al., 2018). Previously, we isolated LDs from BAT and found a tight contact between these two organelles (Yu et al., 2015). A recent study reported that LD-associated mitochondria can be separated from LDs by centrifugation at 9,000 ×*g* and termed them peridroplet mitochondria (PDM) (Benador et al., 2018). To reveal the nature of the interaction and avoid the impacts of housing temperature, we raised mice at their thermoneutral temperature (30 °C) and room temperature (23 °C), and analyzed their BAT. H&E staining demonstrated that most brown adipocytes from the 30 °C group were similar to white adipocytes with a unilocular LD (Fig. 1A, a and b) rather than multilocular LDs that are possessed in BAT at 23 °C (Fig. 1A, d and e). There was a clear change in mitochondrial morphology from an elongated form at 30 °C (Fig. 1A, c) to sphere-shaped at 23 °C (Fig. 1A, f). More importantly, physical contact between LDs and mitochondria was still detected in the 30 °C BAT (Fig. 1A, c, arrows) with no obvious differences in the amount of contact seen in the 23 °C BAT (Fig. 1A, f, arrows).

**Figure 1:**
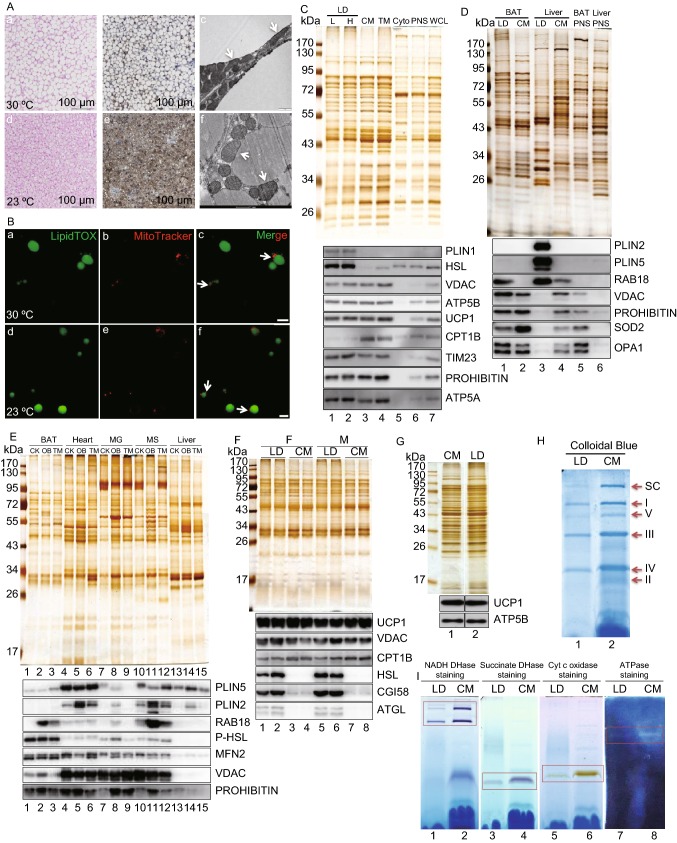
**Lipid droplets and mitochondria form an anchored complex in oxidative tissues**. Eight-week-old male C57BL/6 mice raised at 23 °C were transferred to 30 °C or maintained at 23 °C for one month. **A** Representative H&E staining (a, b), UCP1 immunohistochemistry (c, d), and transmission electron microscopy (TEM) (e, f) of BAT. Arrows point to the physical contact between LDs and mitochondria. **B** Isolated BAT LDs were double stained with LipidTOX Green (a, d) for LDs and MitoTracker Red (b, e) for mitochondria by the method developed in our previous work. Arrows point to the physical contact between LDs and mitochondria in isolated BAT LDs after a merge of the signals. Bar = 5 μm. **C** Eight-week-old male C57BL/6 mice raised at 23 °C were transferred to 30 °C for one month. Isolated BAT LDs (L, 2,000 ×*g*) were subjected to ultracentrifugation (H, 228,000 ×*g*) for 20 min. Equal amounts of protein from the LD, CM (cytosolic mitochondria), TM (total membrane), Cyto (cytosol), PNS (post-nuclear supernatant), and WCL (whole cell lysate) were separated by SDS-PAGE. The gels were subjected to silver staining and Western blot. PLIN1 and HSL represented the enrichment of LD fraction (lanes 1 and 2). The similarity of protein composition between CM (lane 3) and TM (lane 4) suggests that mitochondrion is the major membrane-bound organelle in brown adipocytes. **D** Eight-week-old male C57BL/6 mice were raised at 23 °C. Their BAT and liver were collected for the isolation of LDs, CM, and PNS. The extracted proteins from those cellular fractions were analyzed by silver staining and Western blot. PLIN2 and PLIN5 indicate the enrichment of liver LDs (lane 3) while PLIN2 is not detected and PLIN5 shows a weak signal in BAT LD fraction (lane 1), which is in agreement with previous studies. Therefore, Rab18 here is used as a LD marker protein. **E** Normal monkeys (CK), Obese monkeys (OB), and type 2 diabetes mellitus monkeys (TM) were raised in the native environment. Their BAT, heart, musculus gastrocnemius (MG), musculus soleus (MS), and liver were collected and LDs were isolated from those tissues using ultracentrifugation (40,000 ×*g*) based on the method that we established previously. The extracted proteins from those LD fractions were then analyzed by silver staining and Western blot. LD protein compositions within a tissue were similar among CK, OB and TM monkeys (lanes in each tissue) but clearly varied between tissues (lanes 1/BAT, 4/heart, 7/MG, 10/MS, and 13/liver). Similar to mouse BAT at 23 °C, PLIN2 was not detected and PLIN5 had weak signal (lanes 1–3). In other 4 tissues, PLIN2 could be detected and significantly higher in OB monkey (lanes 5, 8, 11, and 14), while PLIN5 expression level was more consistent. Regarding mitochondrial proteins, liver LDs did not have detectable signals. VDAC was much lower in BAT LDs compared to heart and muscle (lanes 1–3). **F** Ten-week-old female/male C57BL/6 *Plin5*^−/−^ mice were raised at 23 °C, BAT LDs and CM were isolated, and their proteins were analyzed by silver staining and Western blot. LDs were represented by HSL, CGI58, and ATGL (lanes 1, 2, 5, 6). **G** Ten-week-old male 129/SvEv *Plin1*^−/−^ mice were raised at 23 °C. BAT LDs and CM were isolated and their proteins were analyzed by silver staining and Western blot. **H** and **I** Isolated LDs and CM from mouse BAT at 23 °C were treated with digitonin and DDM (9:1, mol/mol). Samples were separated by Blue Native-PAGE and analyzed by Colloidal blue staining (H). Arrows point OXPHOS complex and the gel was also used to indicate protein loading, showing that protein concentration of OXPHOS complexes in LDs (H, lane 1) was lower than in CM (H, lane 2). In-gel enzymatic assay showed the activities of OXPHOS complex I, II, IV and V in LD and CM factions. The red frames represent the in-gel enzymatic activities, including NADH DHase (I, lanes 1 and 2), succinate DHase (I, lanes 3 and 4), Cyt c oxidase (I, lanes 5 and 6), and ATPase (I, lanes 7 and 8). The LDAM contained lower enzymatic activities since the protein loading of LDAM was less than CM (H, lanes 1 and 2)

BAT LDs were then isolated and stained with LipidTOX Green for LDs (Fig. 1B, a and d) and MitoTracker Red for mitochondria (Fig. 1B, b and e) using our previously established methods (Yu et al., 2015). The MitoTracker signals were detected on the LipidTOX-stained spherical structures in isolated LDs from both 30 °C and 23 °C BAT (Fig. 1B, c and f, arrows), further confirming that the contact between LDs and mitochondria exists in mouse BAT even persists at 30 °C. Thus, we isolated LDs from mice housed at 30 °C using two centrifugal forces, 2,000 ×*g* (L) and 228,000 ×*g* (H), to determine if the contact between LDs and mitochondria could be broken by centrifugation as previously reported (Benador et al., 2018). Proteins from the cellular fractions were analyzed by silver staining (Fig. 1C, upper panel) and Western blotting (Fig. 1C, lower panel). Silver staining result presented that the protein profiles of LDs obtained by both centrifugations and cytosolic mitochondria (CM) were almost identical (Fig. 1C, upper panel, lanes 1–3). This similarity was confirmed by Western blotting in which mitochondrial proteins were detected at the same level between the two LD preparations and the isolated mitochondria, including VDAC, ATP5B, UCP1, TIM23, PROHIBITIN, and ATP5A (Fig. 1C, lower panel, lanes 1–3). These findings demonstrated that the contact between LDs and mitochondria in brown adipocytes existed at thermoneutral temperature, and that LD-anchored mitochondria (LDAM) could not be separated from LDs by high centrifugal force (228,000 ×*g*). These studies suggest that the tight contact between these two organelles is a native property of brown adipocytes.

The tight contact/anchoring between LDs and mitochondria was also detected in our previous studies (Li et al., 2016; Zhang et al., 2011), and was confirmed by the analysis of heart LD (Fig. S1A) and skeletal muscle LD (Fig. S1B) proteomes. To determine if the anchoring is restricted to oxidative tissues, mouse liver LDs were isolated and analyzed using the same methods. Figure 1D shows that liver LD fraction had a protein profile distinct from isolated mitochondria (Fig. 1D, upper panel, lanes 3 and 4) and lacked mitochondrial functional proteins (Fig. 1D, lower panel, lane 3). We further conducted similar experiments using rhesus monkey (*Macaca mulatta*) tissues. In agreement with the mouse model, LDs isolated from rhesus monkey BAT, heart, and muscles contained mitochondrial proteins but liver LDs did not (Fig. 1E). LD proteins of control (CK), obese (OB), and diabetic (TM) monkeys were variable, while LDAM proteins were consistent (Fig. 1E, lower panel). These results suggest that LDAM exist specifically in oxidative tissues in mice, rats, and monkeys.

Since PLIN5 was a possible linker between the two organelles, especially in BAT (Benador et al., 2018; Olzmann and Carvalho, 2018), LDs were isolated from BAT of *Plin5*-deficient mice and analyzed. The LD protein profile was similar to the CM (Fig. 1F, upper panel) and mitochondrial proteins were detected at the same level in LDs (Fig. 1F, lower panel). PLIN1 also plays a role in the binding of mitochondria to LDs (Olzmann and Carvalho, 2018). Thus, the same experiments were conducted using *Plin1* knockout mice. The LD fraction from BAT contained a protein composition similar to the isolated mitochondria, demonstrating that PLIN1 was not essential for the anchoring (Fig. 1G). Therefore, neither PLIN1 nor PLIN5 is essential for the connection between LDs and mitochondria to form LDAM. In addition, mild treatment of isolated LDs using Triton X-100, ATP + EGTA, or trypsin could not separate LDAM from LDs (Fig. S2A–D). In particular, the isolated LDs were incubated in the presence or absence of Triton X-100, subjected to centrifugation at 20,000 ×*g* or 265,000 ×*g*, re-isolated and analyzed. Both silver staining and Western blotting demonstrated that LDAM remain no change (Fig. S2B), further confirming that LDs and mitochondria were anchored tightly.

To determine if LDAM are functional mitochondria, the electron transport chain (ETC) and its activity were analyzed using isolated LDs and CM. The mitochondrial ETC complexes were extracted from both LD and CM fractions using detergents, and were separated by blue native gel (BNG) (Figs. 1H and S3). The LDs contained the same ETC complex proteins as the CM, including complexes I, V, III, IV, II (Fig. 1H, arrows). In-gel activity (IGA) was measured and there were comparable enzymatic activities in the LD and mitochondrial fractions (Fig. 1I, rectangles). These data demonstrate that LDAM maintained OXPHOS complexes and had the same enzymatic activities as CM, suggesting that the LDAM were intact and functional.

The anchoring persisted at the thermoneutral temperature of 30 °C (Fig. 1C, lanes 1 and 2), suggesting that it was a native property of brown adipocytes. Therefore, a pre-brown adipocyte cell line (BFC) was used to determine if the anchoring forms during brown preadipocyte differentiation. Figure 2A shows the increased number and size of LDs during the BFC differentiation, and changes in gene expression were presented in Fig. S4A–C. Changes in protein profile were examined by silver staining (Fig. S4D, upper panel) and by Western blot (Fig. S4D, lower panel). The increased expression of UCP1 and PLIN1 further demonstrated that the differentiation of brown adipocytes was successful (Fig. S4D, lower panel). We then isolated LDs from these cells at different stages of differentiation and determined at which point the anchoring between mitochondria and LDs was generated. To increase and enlarge LDs without inducing differentiation, BFC cells were treated with oleic acid (OA) and expression of metabolism-related gene was monitored. No upregulation of UCP1 indicates lack of differentiation (Fig. S4F). Both silver staining (Fig. 2B, upper panel, lanes 1 and 2) and Western blot (Fig. 2B, lower panel, lanes 1 and 2) showed an increase of PLIN2. Mitochondrial proteins were not detectable in LDs isolated from OA-treated cells (Fig. 2B, lower panel, lane 2), suggesting that the increase in LD number and size alone was not sufficient to form the anchoring between LDs and mitochondria in BFC.

**Figure 2:**
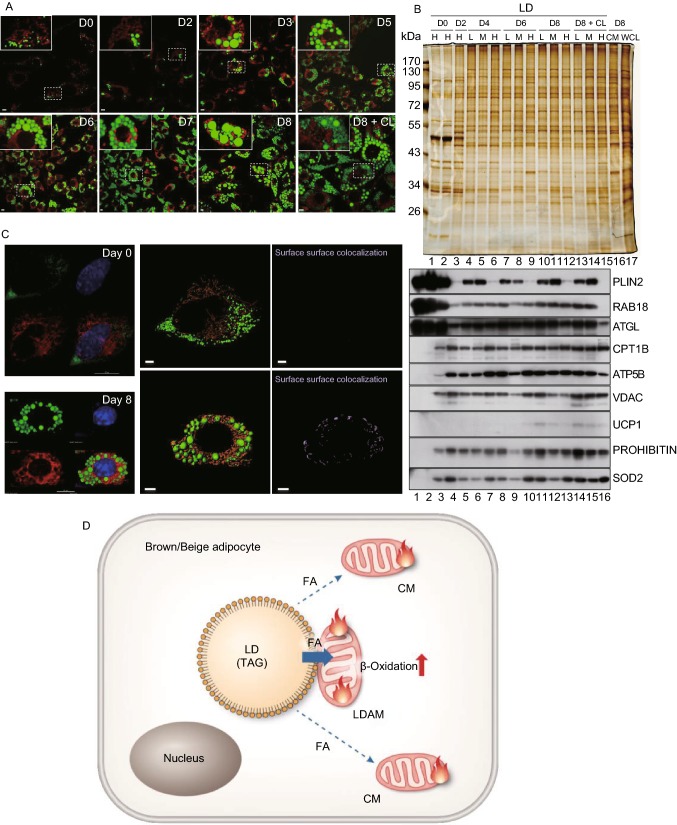
**The anchored lipid droplets and mitochondria are generated during brown adipocyte differentiation**. Brown preadipocytes (BFC) cultured to 100% confluence were induced with induction medium for two days (day 1–2). The medium was changed to differentiation medium for six days (day 3–8). In addition, the brown preadipocytes were treated with oleic acid (OA) (100 μmol/L) for 12 h and the differentiated cells were treated with CL 316,243 (1 μmol/L) at day 8 (D8) for 4 h. LDs were then isolated from these cells, and their proteins were separated using SDS-PAGE and were either stained by silver staining or analyzed by Western blotting. **A** LDs and CM in BFCs at various stages of differentiation were stained with LipidTOX Green and MitoTracker Red, respectively. Bar = 5 μm. D represents the days of differentiation. Result in D8 + CL showed that treatment of CL resulted in smaller LDs, indicating that the cells were differentiated well. **B** LDs were isolated under different centrifugation conditions (Low speed, 2,000 ×*g*; Middle speed, 8,000 ×*g*; High speed, 247,000 ×*g*) from BFCs without any treatment (lane 1), with OA treatment (lane 2), with induction and differentiation at different stages (D2, D4, D6, D8, and D8 + CL) (lanes 3-15). At the same time, CM in BFCs at day 8 were isolated and the whole cell lysate was collected as control (lane 16, lane 17). PLIN2, RAB18, and ATGL were used as LD marker proteins. Differentiation significantly reduced expression of PLIN2, RAB18, and ATGL (lanes 1 and 4). LDs from higher speed centrifugation contained more PLIN2 (for example, lane 4 vs. 5 and 6). **C** LDs, mitochondria, and nuclei in BFCs at day 0 and differentiation day 8 were stained with LipidTOX Green, MitoTracker Red and Hoechst (blue), respectively. Then the images were analyzed by three-dimensional structured illumination microscopy (3D-SIM), and Imaris analysis was applied for the surface-surface colocalization. Bar = 5 μm. **D** Based on previous studies and our current work, we speculate that some mitochondria in brown adipocytes are anchored on LDs, which we term as LDAM. Fatty acids derived from LDs can be transferred into LDAM directly undergoing β-oxidation for making heat, which permits a rapid response to cold challenge

From differentiation day 0 (D0) to D4, mitochondrial proteins in the LD fraction increased gradually, reaching a plateau at D4 (Fig. 2B, lanes 1, 3 and 4–6). On D4, LD protein profiles and the indicated mitochondrial proteins in isolated LDs from three different centrifugation speeds (L, 2,000 ×*g*; M, 8,000 ×*g*; H, 247,000 ×*g*) were similar to each other and also similar to CM (Fig. 2B, lanes 4–6 and 16), suggesting that the two organelles formed tight contact during the BFC differentiation. The anchoring was initiated during early differentiation even without UCP1 expression and, importantly, ultracentrifugation could not break the interaction. In addition, three LD specific proteins, PLIN2, RAB18, and ATGL were significantly reduced from D0 to D4 (Fig. 2B, lower panel, lanes 1 and 4–6).

This contact was also visualized by confocal microscopy. The surface-surface colocalization was almost undetectable in D0 cells (Fig. 2C, upper panel; Video S1). After 8 days of differentiation, surface-surface colocalization was clearly formed between LDs and mitochondria (Fig. 2C, lower panel; Video S2). Pearson correlation analysis (softWoRx6.1.1) was also applied to quantify the degree of colocalization between LDs and mitochondria in BFCs. The Pearson correlation coefficient was higher in differentiated cells at D8 (Fig. S4G, lower panel) than in OA-treated brown adipocytes (Fig. S4G, upper panel), further validating the interaction between LDs and mitochondria in differentiated brown adipocytes. Similar results were obtained from beige adipocyte differentiation (Fig. S4H and S4I). Figure S4H represented differentiation of beige cells and Fig. S4I showed that isolated LDs from differentiated beige cells contained mitochondrial proteins.

Physical contact between LDs and mitochondria may be in two forms, permanent and dynamic. In current understanding, the contact between cellular organelles is dynamic, fitting a “kiss-and-run” model. Based on the discovery of Rab proteins on LDs (Fujimoto et al., 2004; Liu et al., 2007; Liu et al., 2004; Wu et al., 2014) and other early studies, we proposed the hypothesis of multi-recognition sites through which LDs interact with almost all other membrane-bound organelles (Zehmer et al., 2009). The interaction between LDs and mitochondria may also be mediated by PLIN1 and PLIN5 (Olzmann and Carvalho, 2018). These models speculate that lipids and other molecules are exchanged between LDs and the other cellular organelles. Many studies have provided experimental support for these hypotheses (Pu et al., 2011; Szymanski et al., 2007; Valm et al., 2017). In this study, we found that at least in three oxidative tissues LDs and mitochondria are anchored together and may form a permanent complex. These three tissues utilize FAs to produce heat and ATP. But hydrophobic FAs are not only difficult to transport in aqueous cytoplasm but also toxic to the cells. Therefore, we term LDAM and propose the hypothesis that the two organelles are permanently bound as a complex to facilitate FA transport and, in particular, for early response to cold stress in brown adipocytes (Fig. 2D).

## Electronic supplementary material

Below is the link to the electronic supplementary material.

Supplementary material 1 (DOCX 51 kb)

Supplementary material 2 (AVI 159 kb)

Supplementary material 3 (AVI 223 kb)
