# Correction to: Lipid droplets and mitochondria are anchored during brown adipocyte differentiation

**DOI:** 10.1007/s13238-020-00800-z

**Published:** 2021-02-12

**Authors:** Liujuan Cui, Mirza Ahmed Hammad, Shuyan Zhang, Bin Liang, Pingsheng Liu

**Affiliations:** 1 School of Life Sciences, University of Science and Technology of China, Hefei, 230027 China; 2 National Laboratory of Biomacromolecules, CAS Center for Excellence in Biomacromolecules, Institute of Biophysics, Chinese Academy of Sciences, Beijing, 100101 China; 3 University of Chinese Academy of Sciences, Beijing, 100049 China; 4 State Key Laboratory of Conservation and Utilization of BioResources in Yunnan, and Center for Life Sciences, School of Life Sciences, Yunnan University, Kunming, 650091 Yunnan China

## Correction to: Protein Cell 2019, 10(12):921–926 https://doi.org/10.1007/s13238-019-00661-1

The authors would like to update the supplementary information in the published original version. The updated supplementary material is provided in this correction.

## Electronic supplementary material

Below is the link to the electronic supplementary material.

Supplementary file1 (PDF 1110 kb)

