# Supplementary material for: Lipid droplets and mitochondria are anchored during brown adipocyte differentiation
Source: Protein Cell. 2019 Oct 30;10(12):921–6. doi: 10.1007/s13238-019-00661-1 (PMC6881423; doi:10.1007/s13238-019-00661-1)
Supplement: Supplementary file 1 — Supplementary material 1 (DOCX 51 kb) [file 13238_2019_661_MOESM1_ESM.docx]

**Lipid Droplets and Mitochondria Are Anchored during Brown Adipocyte Differentiation**

Liujuan Cui^1,2^, Mirza Ahmed Hammad^2,3^, Shuyan Zhang^2^, Bin Liang^4^, and Pingsheng Liu^1,2,3*^

^1^School of Life Sciences, University of Science and Technology of China, Hefei, Anhui 230027, China

^2^National Laboratory of Biomacromolecules, CAS Center for Excellence in Biomacromolecules, Institute of Biophysics, Chinese Academy of Sciences, Beijing 100101, China

^3^University of Chinese Academy of Sciences, Beijing 100049, China

^4^State Key Laboratory of Conservation and Utilization of Bio-Resources in Yunnan, and Center for Life Sciences, School of Life Sciences, Yunnan University, Kunming 650091, China

*Corresponding author: Pingsheng Liu, E-mail: pliu@ibp.ac.cn

Running title: Lipid Droplets and Mitochondria Form an Anchored Complex

**Supplemental information:**

**Materials and methods**

*Materials*

The Colloidal blue stain kit, TRIzol reagent, HCS LipidTOX^TM^ Green Neutral Lipid Stain (H34475), HCS LipidTOX^TM^ Red Neutral Lipid Stain (H34476), MitoTracker^TM^ Red CMXRos (M7512), PageRuler prestained protein ladder, and SYBR SELECT MASTER MIX were from Thermo Fisher Scientific. Potassium ferrocyanide, 3-Isobutyl-1-methylxanthine, Indomethacin, Digitonin, Dexamethasone, n-Dodecyl b-Dodecyl maltoside, Dimethyl sulfoxide, and CL 316, 243 hydrate were from Sigma-Aldrich. 25% Glutaraldehyde solution, EMbed 812 kit, Uranyl acetate and Lead citrate were purchased from Electron Microscopy Sciences. Osmium tetraoxide (EM grade) was from Nakalai Tesque. Anti-PLIN1 was a gift from Dr. Guoheng Xu. Anti-ATGL was from Cell Signaling Technology. Antibodies against TIM23 and OPA1 were from BD Biosciences. Antibodies against RAB18, CGI58, ACSL1, ACSL5, and CPT1B were from Abclonal Technology. Antibodies against HSL, p-HSL, ATP5A, and MFN2 were from Bioworld Technology. Antibodies against ATP5B, ADRP, total OXPHOS, and UCP1 were from Abcam. Anti-SOD2 antibody was obtained from Proteintech. Anti-VDAC and anti-PROHIBITIN antibodies were obtained from Millipore. Antibody against TOM20 was from Santa Cruz Biotech. Western Lightning Plus ECL was from PerkinElmer. The brown preadipocyte line was a gift from Dr. Patrick Seale. The preadipocytes for beige cell differentiation was a gift from Dr. Xiaodan Lu.

*Animal Studies*

Rhesus monkeys (*Macaca mulatta*) were raised at Kunming Institute of Zoology, Chinese Academy of Sciences.

Eight-week-old male C57BL/6 mice were purchased from Vital River Laboratories (VLR) (Beijing). Chow diet was purchased from Beijing Keao Xieli Feed Co., Ltd. The animals were maintained on 12 h:12 h light-dark cycles. The mice were randomly divided into 3 groups, and were then raised in incubators at different temperatures (30ºC, 23ºC, 6ºC). The mice were allowed free access to standard rodent chow and water. All animal protocols were approved by the Animal Care and Use Committee of the Institute of Biophysics and University of Chinese Academy of Sciences under the permission number SYXK（Jing）2016-0026. Ten-week-old male 129/SvEv Plin1-/- mice were gifts from Dr. Guoheng Xu. Ten-week-old female/male C57BL/6 Plin5-/- mice were gifts from Dr. Jing Ye.

*Isolation of LDs from Mouse Brown Adipose Tissue and LipidTOX Staining*

LDs were isolated by a modified method (Ding et al., 2013; Liu et al., 2004; Yu et al., 2015). Since brown adipocytes have very large LDs and little cytosol, we used a low-speed centrifugation to isolate LDs from brown adipose tissue (BAT). First, we took the interscapular BAT from C57BL/6 male mice into ice-cold NaCl (0.9%) with 0.5 mM PMSF and discarded any attached white fat tissue (WAT). Then the BAT was cut into 1-2 mm^3^ pieces and transferred into 2.5 mL ice-cold Buffer A (20 mM Tricine, pH 7.6, 250 mM Sucrose) with 0.5 mM PMSF. The minced BAT was homogenized by passing through a 200-mesh screen, then the whole cell lysate (WCL) was collected and incubated on ice for 20 min. Then the WCL was centrifuged at 2,000*g* (Low speed, L) for 6 min at 4ºC. The LD fraction on the top was collected into a new 1.5 mL microcentrifuge tube. The remaining supernatant was subjected to ultracentrifugation (Optima^TM^ Ultracentrifuge TLA 100.3) at 303,475*g* (High speed, H) for 15 min. After centrifugation, the middle clear supernatant was collected as cytosol (Cyto) and the pellet was collected as total membrane (TM). LDs were washed with 200 μL Buffer B (20 mM HEPES, pH 7.4, 100 mM KCl, 2 mM MgCl_2_) three times. Finally, 800 μL acetone and 300 μL chloroform was added to the LDs followed by thorough vortexing. Then the mixture was centrifuged at 21,130*g* for 10 min, and the pellet was LD proteins. The LD proteins were dissolved with 2╳ SDS sample buffer and denatured at 95ºC for 5 min for silver staining and Western blot analysis.

Isolated LDs were resuspended with Buffer B and stained with LipidTOX Green/Red on ice for 20-30 min (dilution 1:500, v/v). The LDs were then visualized using an Olympus FV1200 Imaging System.

*Washing and Protease Protection Assay of Isolated LDs*

Isolated BAT LDs (2,000*g*) were centrifuged with ultracentrifugation at 227,943*g* (Optima^TM^ Ultracentrifuge TLA 100.3) for 15 min. Isolated BAT LDs were treated with different concentrations of Triton X-100 and were incubated on ice for 10 min. In another experiment, isolated BAT LDs were treated with ATP and EGTA and were incubated at room temperature for 10 min. Besides, isolated BAT LDs were also treated with different concentrations of trypsin and were incubated at 37ºC for 10, 60, and 120 min, respectively. During the reactions, the tubes were flicked every 5 min. After incubation, LDs were re-isolated by centrifugation at 2,000*g* at 4ºC for 6 min. The solution and pellet were discarded and the LDs were washed twice with Buffer B. LD proteins were extracted by acetone and chloroform and dissolved with 2╳ SDS sample buffer. The samples were kept for further analysis.

*Isolation of Mitochondria from Mouse Brown Adipose Tissue and MitoTracker Staining*

Mitochondria were isolated by a modified method (Pu et al., 2011; Yu et al., 2015). WCL was centrifuged at 500*g* for 10 min to remove the nucleus and the supernatant was collected as the post-nuclear supernatant (PNS). The PNS was centrifuged at 8,000*g* for 10 min at 4ºC. The pellet was resuspended and washed twice with 500 μL Buffer B as crude mitochondria. The mitochondrial fraction was loaded carefully on the top of a Percoll step gradient (3 mL 50% and 8 mL 25%) and was centrifuged at 18,000 rpm (Optima^TM^ L-100 XP Ultracentrifuge) for 45 min at 4ºC. The interface between 50% and 25% Percoll was collected (200 μL) into a new tube. The collected fraction was diluted by the addition of 1 mL Buffer B, vortexed thoroughly, and centrifuged at 21,130*g* for 10 min. The pellet was washed three times with 1 mL Buffer B to remove Percoll.

Isolated mitochondria were resuspended with Buffer B and stained with MitoTracker Red on ice for 20 min (dilution 1:1000, v/v). The samples were visualized with an Olympus FV1200 Imaging System.

*Cell Culture and Differentiation*

Brown preadipocytes (BFCs) were grown in Dulbecco’s Modified Eagle Medium/Nutrient Mixture F-12 (DMEM/F12) (Gibco, Life Invitrogen) containing 10% Fetal Bovine Serum (FBS) (Gibco, Life Invitrogen) and 1% Penicillin/Streptomycin. BFC differentiation was induced by incubating cells with induction medium containing 20 nM Insulin, 1 nM Triiodothyronine (T3), 0.5 mM 3-Isobutyl-1-methylxanthine (IBMX), 1 μM Dexamethasone (Dex), and 125 µM Indomethacin (Indo) for two days (days 1-2). On the following days, the medium was replaced with differentiation medium containing 20 nM Insulin, 1 nM Triiodothyronine (T3) with a change of medium every two days (days 3-8) (Uldry et al., 2006). As a control, BFCs were treated with Sodium Oleate (OA) (100 µM) in growth medium for 12 h.

*Confocal Microscopy*

BFCs at different stages were incubated in medium containing 50 nM MitoTracker Red at 37ºC for 30 min. The medium was discarded and the cells were incubated in medium containing LipidTOX Green (dilution 1:1000, v/v) and Hoechst 33258 (dilution 1:1000, v/v) at 37ºC for 30 min. Fluorescence signals were captured using an Olympus FV1200 Imaging System and three-dimensional structured illumination microscopy (3D-SIM). Surface-surface colocalization was analyzed by Imaris 8.1.2. Pearson correlation analysis (softWoRx6.1.1) was applied to quantify the degree of colocalization between LDs and mitochondria.

*Electron Microscopy*

Through ultra-thin sectioning, the ultra-structure of BAT was examined by transmission electron microscopy (TEM). Briefly, after collected and rinsed, the BAT tissue was cut into small pieces (about 1 mm^3^). These pieces were fixed in 2.5% (w/v) Glutaraldehyde in 0.1 M PB (pH 7.2) for 2 h at room temperature. Subsequently, they were fixed in 1% (w/v) Osmium Tetraoxide (with 1% Potassium Ferrocyanide) for 2 h. After dehydrated in an ethanol series at room temperature, the samples were embedded in EMbed 812 and finally prepared as 70 nm ultra-thin sections. After stained with uranyl acetate and subsequently with lead citrate at room temperature, the sections were observed with Tecnai Spirit electron microscope.

The isolated LDs and mitochondria were also examined by TEM. Briefly, isolated LDs were embedded in 4% agarose first. After solidification, the samples were cut into small blocks (~1 mm^3^). The pellets of isolated mitochondria were fixed directly. The samples were prefixed in 1% Glutaraldehyde for 30 min and then post-fixed in 1% Osmium Tetraoxide (with 1% Potassium Ferrocyanide) for 30 min at room temperature. The samples were finally prepared as 70 nm sections and viewed with electron microscope.

*Isolation of LDs from Brown Adipocytes*

LDs were isolated from brown adipocytes according to the protocol previously described (Ding et al., 2013; Liu et al., 2004). Briefly, cultured brown adipocytes were washed twice with ice-cold NaCl (0.9%) and were then collected by scraping into ice-cold NaCl (0.9%). The cells were pelleted by centrifugation at 1,000*g* for 10 min at 4ºC, resuspended in Buffer A (20 mM Tricine, 250 mM Sucrose, pH 7.8) containing 0.5 mM PMSF, and kept on ice for 20 min. The cell suspension was pressurized to 700 psi with N_2_ gas and held on ice for 20 min, followed by homogenization with cavitating. The homogenate was centrifuged at 1,000*g* for 10 min at 4ºC and the supernatant was transferred into SW 40 Ti tubes. Then Buffer B (20 mM HEPES, 100 mM KCl, 2 mM MgCl_2_, pH 7.4) was loaded onto the top of the supernatant and the gradient was subjected to ultracentrifugation (Optima^TM^ L-100 XP Ultracentrifuge) at the speed (Low speed, 2,000*g*; Middle speed, 8,000*g*; High speed, 247,000*g*) and time indicated at 4ºC. After each centrifugation, LDs were carefully collected in a minimal volume using a 200 µL pipette tip. The LDs were then washed three times with Buffer B and the LD proteins were extracted with 800 μL acetone and 300 μL chloroform for further analysis.

*Silver Staining and Western Blotting*

For the silver staining, the gel was fixed in fixation buffer for 30 min and then incubated in sensitization buffer at room temperature for 30 min. The gel was then washed four times with ddH_2_O for 5 min each time. The washed gel was treated with silver staining buffer at room temperature for 20 min, followed by developing buffer until the bands appeared. The reaction was blocked in stopping buffer immediately after achieving satisfactory resolution.

For Western blotting, proteins from different fractions were separated by sodium dodecyl sulfate-polyacrylamide gel electrophoresis (SDS-PAGE) and were transferred to PVDF membranes. The membranes were blocked with 5% BSA at room temperature for 1 h and were then incubated with primary antibodies at room temperature for 1 h or 4ºC overnight. The membranes were washed 3 times with washing buffer for 5 min each time followed by incubation in the indicated secondary antibodies at room temperature for 1 h. The protein bands were visualized with Western Lightning Plus ECL.

*Quantitative Real-Time PCR*

Total RNA was extracted with TRIzol reagent and reverse transcribed into cDNA according to the manufacturer’s protocol (Promega and TAKARA). qRT-PCR was performed using SYBR SELECT MASTER MIX and Biosystems QuantStudio 7 Flex for 40 cycles and the fold change for all the samples was calculated by the 2-ΔΔCt method, n ≥ 6. β-actin was used as housekeeping gene for mRNA expression analysis.

*Blue Native Gel Electrophoresis and In Gel Activity*

Blue native gel electrophoresis was performed as previously described with minor modifications (Wittig et al., 2006). The resuspended LDs and mitochondria were solubilized with different proportions of Digitonin and n-Dodecyl-β-D-Maltoside (DDM) (10:1, 9:1, 8:2, mol/mol) for 15 min. After the reaction, the samples were centrifuged at 21,130*g* for 30 min and the supernatant was collected and mixed with glycerol and Coomassie blue G-250. Finally, the samples were separated using 4%-13% acrylamide gradient gels.

For the in gel activity assay, the activities of respiratory complexes were analyzed as previously described with some modifications (Khvorostov et al., 2008). (i) Complex I activity: the blue native gels (BNGs) were incubated in the reaction buffer with 5 mM Tris-Cl, pH 7.4, 1 mg/mL Nitro Blue Tetrazolium (NBT) and 0.14 mM NADH at room temperature. (ii) Complex II activity: the BNGs were incubated in the reaction buffer with 50 mM Phosphate buffer, pH 7.4, 84 mM Succinic Acid, 0.2 mM Phenazine Methosulfate, 2 mg/mL NBT, and 4.5 mM EDTA. (iii) Complex IV activity: the BNGs were incubated in 5 mg Diaminobenzidine Tetrahydrochloride (DAB) dissolved in 9 mL of 50 mM Phosphate buffer, pH 7.4, containing 10 mg Cytochrome c and 750 mg Sucrose. (iv) Complex V activity: the BNGs were incubated in 50 mM Glycine for 1 h after washing three times with ddH_2_O. Then the BNGs were incubated in reaction buffer with 35 mM Tris, 270 mM Glycine, 14 mM MgSO_4_, 5 mM ATP, 0.2% (w/v) Pb(NO_3_)_2_, pH was adjusted to 7.8. The gels were incubated in the solution at 37°C. All reactions were stopped when the bands appeared.

**Supplemental figures:**

**Figure S1 The Tight Contact between Lipid Droplets and Mitochondria in Heart and Muscle**

Rat heart LD (Li et al., 2016) and mouse skeletal muscle LD (Zhang et al., 2011) proteomes were mapped for mitochondrial proteins using MitoCarta2.0 (Calvo et al., 2016). The analysis identified 236 mitochondrial proteins in rat heart LDs out of a total of 752 proteins. The mouse skeletal muscle LD proteome contained 96 mitochondrial proteins out of 324 proteins. Keyword-based pathway clustering was performed for the rat heart LD proteome (A) and mouse skeletal muscle LD proteome (B). Many proteins in both proteomes were involved in major mitochondrial pathways.

**Figure S2 The Tight Contact between Lipid Droplets and Mitochondria in Mouse BAT**

**A** Eight-week-old male C57BL/6 mice were raised at 23°C. BAT LDs were isolated, and then distributed into three aliquots. The aliquots were treated with Triton X-100 on ice for 10 min at the concentration (%, v/v) indicated. After incubation, LDs were re-isolated, the pellets were collected, and the proteins were analyzed by silver staining. Very small amount of proteins was in the pellet (lanes 2 and 3 vs. 4 and 5), suggesting that LDAM could not be stripped by 0.025% Triton X-100. **B** Eight-week-old male 129/SvEv *Plin1*^-/-^ mice were raised at 23ºC. Their BAT LDs were isolated and distributed into four aliquots. The aliquots were treated with Triton X-100 (%, v/v) on ice for the indicated time. After incubation, LDs were re-isolated using the indicated speed and the extracted LD proteins were analyzed by silver staining and Western blotting. Compared to control (lane 1), both protein profile (upper panel) and indicated proteins (lower panel) did not show much difference, indicating that 0.05% Triton X-100 plus high-speed centrifugation could not separate LDAM from LDs. **C** BAT LDs were isolated from mice housed at 23°C, and were distributed into two aliquots. The aliquots were treated with the indicated concentration of ATP and EGTA at 37°C for 10 min. After incubation, LDs were re-isolated and the pellets were collected. Their proteins and isolated CM proteins were analyzed by silver staining. **D** BAT LDs were isolated from mice housed at 23°C, and were distributed into nine aliquots. The aliquots were treated with the indicated concentration (mg/mL) of trypsin for indicated time at 37°C. After incubation, LDs were re-isolated, and the pellets were collected. The proteins were analyzed by silver staining and Western blotting. After treatment, the LD resident protein PLIN1 and the mitochondrial outer membrane protein TOM20 were completely digested while the mitochondrial proteins UCP1 and ATP5B remained at least to some extent (lower panel). Thus, the proteinase treatment did not sever the connection between LDs and mitochondria. The mitochondria remained intact since UCP1 and ATP5B were protected from trypsin digestion. A previous study reported that PLIN1 is a tethering protein between the two organelles (Olzmann and Carvalho, 2018). However, this is not supported by our result since digestion of PLIN1 did not release mitochondria from LDs.

**Figure S3 Mitochondrial ETC Complexes of Lipid Droplet-anchored Mitochondria**

Eight-week-old male C57BL/6 mice were raised at 23°C. Isolated BAT LDs and CM were treated with different proportions of digitonin and n-Dodecyl-β-D-maltoside (DDM) (10:1, 9:1, 8:2, mol/mol). Samples were separated by Blue Native-PAGE and were analyzed by silver staining. The result showed the mitochondrial OXPHOS complexes, including super complex (SC) and complex I, V, III, IV, II.

**Figure S4 Differentiation of Brown Adipocytes and Beige Cells**

Brown preadipocytes (BFC) and beige precursor cells cultured to 100% confluence were induced with induction medium for two days (day 1-2). The medium was changed to differentiation medium for six days (day 3-8). In addition, 1 μM Rosiglitazone was added in to the medium for beige cell differentiation. LDs were then isolated from these cells, and their proteins were separated using SDS-PAGE and either stained by silver staining or analyzed by Western blotting. **A/B/C** Analysis of metabolic gene expression by qRT-PCR in BFCs with or without induction and differentiation at different stages (D0, D2, D4, D6, and D8). n = 3. Statistical analysis was performed using Student’s t test. *, P<0.05; **, P<0.01; ***, P<0.001. Ucp1 and prdm16 were significantly stimulated, demonstrating the differentiation successful. **D** Proteins at different differentiation stages (Day 0-8) were separated by SDS-PAGE and analyzed by silver staining and Western blotting using the indicated antibodies. UCP1 expression from D6 to D8 represented that the cells were differentiated to brown adipocytes. Other mitochondrial proteins, such as CPT1B, TIM23, and TOM20, remained unchanged during differentiation. **E/F** Brown preadipocytes (BFCs) were treated with oleate (OA) (100 μM) for 12 h. Then the expression of metabolic genes was analyzed using qRT-PCR (E). n = 3. Statistical analysis was performed using Student’s t test. *, P<0.05; **, P<0.01; ***, P<0.001. LDs and CM in BFCs which were treated with or without OA were stained with LipidTOX Green and MitoTracker Red (F), respectively. Bar = 5 μm. Although transcription of *Plin1* and *Cd36* was stimulated, *Ucp1* was not, suggesting that OA did not induce differentiation. **G** LDs and mitochondria in BFCs at day 0 with OA treatment and differentiation day 8 were stained with LipidTOX Green and MitoTracker Red, respectively. Then the images were analyzed by three-dimensional structured illumination microscopy (3D-SIM), and Pearson correlation analysis (softWoRx6.1.1) was applied to quantify the degree of colocalization between LDs and mitochondria. Bar = 5 μm. **H** LDs and CM at various stages of beige cell differentiation were stained with LipidTOX Green, MitoTracker Red, and Hoechst (blue), respectively. Bar = 5 μm. The size of LDs indicated the differentiation stages. **I** LDs (Middle speed, M; Low speed, L) and CM in brown adipocytes (B1) and beige adipocytes (B2) at differentiated Day 8 were isolated and their proteins were analyzed by silver staining and Western blotting. LD fractions for both differentiated brown adipocytes and beige cells contained p-HSL and PLIN1, as well as mitochondrial protein VDAC, suggesting that LDs and mitochondria in beige cells were also anchored.

Calvo, S.E., Clauser, K.R., and Mootha, V.K. (2016). MitoCarta2.0: an updated inventory of mammalian mitochondrial proteins. Nucleic Acids Res *44*, D1251-1257.

Ding, Y., Zhang, S., Yang, L., Na, H., Zhang, P., Zhang, H., Wang, Y., Chen, Y., Yu, J., and Huo, C. (2013). Isolating lipid droplets from multiple species. Nature protocols *8*, 43.

Khvorostov, I., Zhang, J., and Teitell, M. (2008). Probing for mitochondrial complex activity in human embryonic stem cells. Journal of visualized experiments: JoVE.

Liu, P., Ying, Y., Zhao, Y., Mundy, D.I., Zhu, M., and Anderson, R.G. (2004). Chinese hamster ovary K2 cell lipid droplets appear to be metabolic organelles involved in membrane traffic. The Journal of biological chemistry *279*, 3787-3792.

Olzmann, J.A., and Carvalho, P. (2018). Dynamics and functions of lipid droplets. Nature Reviews Molecular Cell Biology, 1.

Pu, J., Ha, C.W., Zhang, S., Jung, J.P., Huh, W.-K., and Liu, P. (2011). Interactomic study on interaction between lipid droplets and mitochondria. Protein & cell *2*, 487-496.

Uldry, M., Yang, W., St-Pierre, J., Lin, J., Seale, P., and Spiegelman, B.M. (2006). Complementary action of the PGC-1 coactivators in mitochondrial biogenesis and brown fat differentiation. Cell Metabolism *3*, 333-341.

Wittig, I., Braun, H.-P., and Schägger, H. (2006). Blue native PAGE. Nature Protocols *1*, 418.

Yu, J., Zhang, S., Cui, L., Wang, W., Na, H., Zhu, X., Li, L., Xu, G., Yang, F., and Christian, M. (2015). Lipid droplet remodeling and interaction with mitochondria in mouse brown adipose tissue during cold treatment. Biochimica Et Biophysica Acta (BBA)-Molecular Cell Research *1853*, 918-928.
